# The purinergic signaling in Alzheimer's disease: possible use of a shed form of the P2X7 receptor (sP2X7R) as a biomarker of early dementia

**DOI:** 10.1002/alz70856_103288

**Published:** 2025-12-24

**Authors:** Simonetta Falzoni, Mario Tarantini, Nelly Redolfi, Dorianna Sandonà, Martina Bedetta, Annamaria Lia, Elisa Greotti, Giovanni Zorzi, Carlo Gabelli, Angelo Antonini, Paola Pizzo, Giulia Musso, Anna Lisa Giuliani

**Affiliations:** ^1^ University of Ferrara, FERRARA, Ferrara, Italy; ^2^ University of Padova, Padova, Padova, Italy; ^3^ CNR Institute of Neuroscience, Padova, Padova, Italy; ^4^ Padua University‐Hospital, Padova, Padova, Italy; ^5^ University of Padova, Padova, Italy

## Abstract

**Background:**

Neuroinflammation, one of the recognised factors in AD, is likely sustained by the presence of high amounts of extracellular ATP (eATP) in the inflammed brain. One main goal of our study was the measurement of eATP levels in the brain of an AD mice model using suitable luminescent probes produced in our laboratory. A main receptor for eATP is the P2X7 receptor (P2X7R), highly expressed by immune cells, which activates the NLRP3 inflammasome with consequent release of inflammatory mediators. We recently found a plasma shed form of the P2X7R (sP2X7R), higher in inflammatory conditions. Here we aimed to verify if the sP2X7R was higher in AD patients.

**Method:**

The pmeLUC probe for eATP was employed via retro orbital (RO) sinus injection in an AD mouse model (B6.152H), expressing human PS2‐N141I and APP Swedish mutations, to measure the eATP brain levels at different mouse ages. The levels of P2X7R, NLRP3 and inflammatory cytokines (IL‐1 beta, IL‐6 and TNF‐alfa) were measured in cortical homogenates. In humans, we measured by ELISA the sP2X7R in plasma samples from three cohorts, previously tested for clinical cognitive parameters: healthy control subjects (HC), subjects with mild cognitive impairment (MCI), and patients with AD.

**Result:**

We detected significantly higher levels of eATP in the brain of 2‐ 6‐ and 9‐month‐old AD mice respect to age‐matched control mice. Significantly, the increase of eATP concentrations was present at 2 months of mouse age as a sign of early inflammation, before Aβ‐plaque brain deposition. Overall, the cytokine profile obtained suggests that neuroinflammation accompanies AD progression and begin before the onset of Aβ plaque deposition. In agreement with these results, in humans, we detected a statistically significant increase in sP2X7R plasma concentrations in MCI patients compared to HC subjects or AD patients where the sP2X7R levels were higher compared to controls, without reaching the significance.

**Conclusion:**

The contribution of eATP in AD neuroinflammation is strongly supported by our results. In addition, the shed form of the P2X7R (sP2X7R) appears a promising biomarker for identifying early forms of cognitive impairment likely progressing to a more serious dementia. (Research funded by the Cure Alzheimer's Fund)

